# Sarcopenia and Frailty in Cirrhotic Patients: Evaluation of Prevalence and Risk Factors in a Single-Centre Cohort Study

**DOI:** 10.3390/medicina61050821

**Published:** 2025-04-29

**Authors:** Kadri Atay, Seval Aydin, Billur Canbakan

**Affiliations:** 1 Department of Gastroenterology, Mardin Research and Training Hospital, Mardin 47100, Türkiye; 2 Division of Biochemistry, Cerrahpasa Faculty of Medicine, İstanbul 34098, Türkiye; seval.aydin@iuc.edu.tr; 3 Division of Gastroenterology, Cerrahpasa Faculty of Medicine, İstanbul 34098, Türkiye; billurcanbakan@yahoo.com

**Keywords:** cirrhosis, sarcopenia, frailty, SARC-F score, CTP score, MELD score

## Abstract

*Background and Objectives*: Sarcopenia and frailty adversely affect morbidity and mortality in patients with liver cirrhosis. This study aimed to investigate the prevalence of sarcopenia and frailty in cirrhotic patients and to identify the contributing factors. *Materials and Methods*: This study was conducted in adult patients diagnosed with cirrhosis in a single-center cohort study who were under follow-up in the gastroenterology outpatient clinic. Patients were evaluated using the SARC-F questionnaire, FRAIL index, handgrip strength measurements, and various biochemical parameters. *Results*: Of the 100 patients included in the study, 58.7% were male, with a median age of 66.5 years. The prevalence of sarcopenia was 32%. Patients with sarcopenia had significantly lower body mass index (BMI) and higher model for end-stage liver disease (MELD)-Na and Child–Turcotte–Pugh (CTP) scores. According to the FRAIL scale, pre-frailty was highly prevalent among patients (60%). Significant negative correlations were observed between the SARC-F score and BMI, handgrip strength, albumin, vitamin D, and sodium levels. Conversely, significant positive correlations were identified between the SARC-F score and age, CTP score, MELD-Na score, bilirubin, AST, ALT, and ferritin levels. *Conclusions*: This study demonstrated a high prevalence of sarcopenia and frailty among cirrhotic patients. These findings warrants further investigation in longitudinal studies for hard clinical outcome and mortality.

## 1. Introduction

Liver cirrhosis is a progressive and often irreversible condition characterized by scarring and deformation of healthy liver tissue as a result of chronic liver diseases. Cirrhosis ranks as the 11th leading cause of death globally [1,2,3]. Common causes of cirrhosis include hepatitis B and C virus infections, alcohol consumption, metabolic dysfunction-associated steatotic liver disease (MAFLD, MASH), and autoimmune diseases [4,5]. Prognostic evaluation in patients with cirrhosis is a critical yet complex process. Commonly used scoring systems, such as the Child–Turcotte–Pugh (CTP) and model for end-stage liver disease (MELD) scores, are valuable tools for predicting mortality in these patients [6,7]. Sarcopenia, defined as a loss of muscle mass due to muscle wasting, is often associated with reduced muscle strength and function [8,9]. In cirrhotic patients, the depletion of functional liver reserves leads to various potential complications, including malnutrition and the development of sarcopenia [10,11,12]. Muscle loss in chronic disease states has been categorized into two types: “primary sarcopenia”, related to aging, and “secondary sarcopenia”, associated with chronic conditions such as liver, heart, lung, and kidney diseases [8,13]. The prevalence of sarcopenia in cirrhotic patients ranges from approximately 23% to 60%, depending on the stage of liver disease and the etiology of cirrhosis at diagnosis [14]. Studies conducted in Turkey have reported prevalence rates between 40% and 55% [15]. Skeletal muscle mass is maintained through a balance between protein synthesis and protein degradation. In cirrhotic patients, factors such as inadequate glycogen stores due to liver atrophy, insufficient energy intake, disruptions in the liver-muscle axis, systemic inflammation, increased muscle autophagy, and low testosterone levels contribute to the development of sarcopenia [16,17]. These catabolic processes increase the utilization of amino acids as an energy source, accelerating their breakdown in skeletal muscle. The early recognition of sarcopenia is crucial for healthcare management in these patients. Consequently, several authoritative guidelines recommend routine screening programs for sarcopenia in cirrhotic patients [18,19]. Frailty, a multifaceted syndrome, reflects increased vulnerability to stressors and is characterized by a gradual loss of capacity across multiple physiological systems, reduced physiological reserves, and functional decline [9,15,20]. Similarly to sarcopenia, frailty is common in cirrhotic patients. However, due to the use of diverse assessment tools and threshold values, accurately reporting the prevalence of frailty is challenging [21]. Sarcopenia and frailty have a prominent effect on the poor clinical outcomes and mortality of liver cirrhosis (LC). However, the role of factors associated with sarcopenia and frailty in this patient population remains insufficiently explained. Therefore, in this single-center, cross-sectional study, we aimed to comprehensively evaluated the prevalence and potentially associated risk factors in cirrhotic outpatients and to clarify contributing variables and identify high-risk groups and facilitate the development of strategies for the early prevention of these conditions.

## 2. Methods

### 2.1. Study Design and Participants

We screened 750 adult patients who regularly followed up at the Gastroenterology outpatient clinic. Patients diagnosed with cirrhosis at least 6 months prior, a willingness to participate in the study, and adequate follow-up data related to the diagnosis of cirrhosis enrolled study. Study included 100 patients aged 18 years or older who were diagnosed with cirrhosis of various etiologies. Patients with severe comorbidities such as renal failure or hepatocellular carcinoma (HCC) or hepatic encephalopathy who did not cooperate during the evaluation were excluded. This study was conducted in accordance with the guidelines set forth in the Declaration of Helsinki, and all procedures were approved by the Clinical Research Ethics Committee of Cerrahpasa University Medical Faculty hospital with decision number 2024-205.

### 2.2. Data Collection

Demographic data (age, sex), anthropometric measurements (height, weight, and body mass index [BMI]), and disease etiologies were recorded. A hepatologist assessed the participants’ clinical status and laboratory parameters using hospital medical records. The severity and progression of liver disease were evaluated using the Child–Turcotte–Pugh (CTP) score [6] and the model for end-stage liver disease (MELD) score [7]. The CTP score was calculated based on ascites, encephalopathy, total bilirubin, albumin levels, and international normalized ratio (INR). The MELD score, a comprehensive scoring system for assessing the severity of chronic liver disease and predicting survival, was derived using serum bilirubin, sodium, creatinine, and prothrombin time values. Complications related to cirrhosis, such as ascites, varices, and encephalopathy, were also evaluated.

*SARC-F Questionnaire*

Strength: How much difficulty do you have lifting and carrying 5 kg?None = 0; Some = 1; A lot or unable = 2Walking Assistance: How much difficulty do you have walking across a room?None = 0; Some = 1; A lot, use aids, or unable = 2Chair Stand: How much difficulty do you have rising from a chair or bed?None = 0; Some = 1; A lot or unable without help = 2Stair Climbing: How much difficulty do you have climbing a flight of 10 stairs?None = 0; Some = 1; A lot or unable = 2Falls: How many times have you fallen in the past year?None = 0; 1–3 falls = 1; 4 or more falls = 2

Each item is scored on a scale from 0 (best) to 2 (worst), with a total score ranging from 0 to 10. Higher scores (SARC-F ≥ 4) indicate a higher risk of sarcopenia [22].

*FRAIL Scale*

Fatigue: Do you experience fatigue or tiredness?Resistance: Can you walk one block without resting?Ambulation: Can you climb stairs?Illness: Do you have more than five medical conditions under follow-up or treatment?Weight Loss: Have you unintentionally lost 5% or more of your body weight in the past six months?

Scoring ranges from 0 to 5, with one point assigned for each positive response. Health status is classified as frail (3–5), pre-frail (1–2), or robust (0) [23].

### 2.3. Evaluation of Hand Grip Strength

After patients were adequately rested, hand grip strength measurement was performed in a sitting position. Before the hand grip strength measurement, the physician demonstrated the correct position and use of the dynamometer to the patients. A Baseline Hydraulic Hand Dynamometer (200 lb) was used for measurement. The measurement was taken with the patient sitting, the shoulder and arm in a neutral position, the elbow flexed at a 90-degree angle, and the forearm extended parallel to the ground while grasping the hydraulic hand dynamometer. Patients were asked to squeeze the dynamometer with all their strength in this position. Three measurements for each hand were recorded, and their averages were noted.

### 2.4. Statistical Analysis

Participant characteristics were summarized as median values (including lower and upper quartiles) for continuous variables and as percentages (%) and numbers (n) for categorical variables. Statistical analyses were performed using the SPSS software package (Windows version 23). Comparisons between the sarcopenia and non-sarcopenia groups were made using the Chi-square and Fisher’s exact tests for categorical variables, and the Mann–Whitney U test for continuous variables. Correlations with the SARC-F score were analyzed using Spearman’s correlation. Spearman’s rank correlation coefficient is significant at *p* < 0.05 and *p* < 0.01. A confidence interval of 95% was used for all analyses, and results with *p* < 0.05 were considered statistically significant.

## 3. Results

Of the 100 patients included in the study, 59 (58.7%) were male. The median age was 66.5 years, and the median BMI was 26 ± 5.4 kg/m². The etiologies of cirrhosis were cryptogenic cirrhosis (21.3%), metabolic-associated steatohepatitis (MASH) (21.3%), hepatitis B (14.7%), alcohol (13.3%), and other factors (29.4%). The majority of the participants were physically active and able to leave their homes (74.7%). According to the Child–Turcotte–Pugh (CTP) classification, 46.7% of the patients were classified as Child A, 28% as Child B, and 25.3% as Child C. The median (interquartile range, IQR) MELD and MELD-Na scores were 12 (9–15) and 13 (9–17), respectively. Ascites was present in 41.3% of the patients, encephalopathy in 32% (24% controlled, 8% uncontrolled), and varices in 64% (49.3% without bleeding, 14.7% with bleeding) (Table 1).

### 3.1. SARC-F Score Distribution

The distribution of SARC-F scores revealed that 25 patients (25.3%) scored 0, 25 (25.3%) scored 1, 10 (9.3%) scored 2, 8 (8%) scored 3, and 32 (32.1%) scored ≥ 4. Table 2 illustrates the distribution of SARC-F scores and the prevalence of sarcopenia, defined as a score of ≥4. In this study, sarcopenia was diagnosed in 32% of patients (32 patients).

No statistically significant difference in sarcopenia prevalence was observed between genders (prevalence in females: 38%, males: 27%; *p* = 0.427). Patients with sarcopenia had significantly lower BMI, higher MELD-Na scores, and higher Child scores compared to those without sarcopenia. Additionally, patients with sarcopenia were more likely to be homebound and had lower grip strength in both hands. The prevalence of sarcopenia according to the Child classification was 29.2% in Child A, 45.8% in Child B, and 25% in Child C (Table 2).

### 3.2. Frailty Prevalence

Frailty, assessed using the FRAIL scale, was prevalent, with pre-frailty observed in 60% of all cirrhotic patients (Table 3). Patients with sarcopenia had significantly lower albumin (30 vs. 38 g/L, *p* = 0.004) and vitamin D levels (16 vs. 32, *p* = 0.008) and higher bilirubin (1.9 vs. 1.1, *p* = 0.01), AST (67 vs. 46, *p* = 0.029), ALT (47.5 vs. 27, *p* = 0.025), and ferritin (49 vs. 35, *p* = 0.032) levels compared to those without sarcopenia (Table 4).

### 3.3. Correlation Analysis

Correlation analysis of SARC-F scores and potential risk factors for sarcopenia revealed statistically significant negative correlations between SARC-F scores and BMI (r = −0.43, *p* < 0.002), grip strength (right hand: r = −0.492, *p* < 0.001; left hand: r = −0.403, *p* < 0.001), albumin (r = −0.344, *p* = 0.003), vitamin D (r = −0.400, *p* = 0.002), and sodium levels (r = −0.437, *p* < 0.001).

In contrast, SARC-F scores were positively correlated with age (r = 0.25, *p* = 0.032), Child score (r = 0.271, *p* = 0.019), MELD-Na score (r = 0.384, *p* = 0.001), bilirubin (r = 0.336, *p* = 0.002), AST (r = 0.245, *p* = 0.024), ALT (r = 0.263, *p* = 0.024), and ferritin (r = 0.287, *p* = 0.013) (Table 5).

A correlation analysis of SARC-F scores and another potential risk factors for sarcopenia revealed statistically no correlations between SARC-F scores and Meld Score, FRAIL score, creatinine, total protein level, GGT, ALP, INR, and hemoglobin.

## 4. Discussion

This study analyzed the prevalence of sarcopenia among 100 patients with cirrhosis and evaluated potential risk factors, including specific anthropometric, biochemical, and frailty-related parameters, that may be associated with sarcopenia in this population.

Sarcopenia is one of the most prominent features of cirrhosis, significantly contributing to increased morbidity and mortality in this patient group [24]. Reported prevalence rates of sarcopenia in cirrhotic patients range from 20% to 70%, markedly higher than the prevalence rates of 2.9–38.5% reported in the general elderly population [25,26]. Studies conducted in Turkey have suggested sarcopenia prevalence rates between 40% and 55% [15]). According to a recent meta-analysis, the prevalence of sarcopenia in cirrhotic patients is approximately 37.5%, with higher rates reported among males, patients with alcohol-related liver cirrhosis, and those with advanced disease stages [27]. Consistently with these findings, we determined the prevalence of sarcopenia in our study as 32%, with no significant difference between genders.

The variability in prevalence rates may stem from differences in diagnostic tools, variations in the severity of liver diseases, and the diverse health profiles of the general population. Although various approaches have been proposed for diagnosing sarcopenia, their routine clinical use is often limited due to cost and time constraints. Multi-center studies have demonstrated the validity of using the SARC-F score as a screening tool to identify chronic liver disease patients at high risk for sarcopenia [28]. In line with the literature, we used the SARC-F score in our study, setting a cutoff value of ≥4, which revealed a sarcopenia prevalence of 32%.

Malnutrition in chronic diseases can be detected using simple, low-cost parameters such as body weight, BMI, and weight changes. However, the presence of fluid retention and ascites in cirrhotic patients may lead to misinterpretation of these parameters. Conversely, other studies have demonstrated that BMI is a reliable parameter for detecting malnutrition in cirrhotic patients [29]. In agreement with previous studies [30,31], we found that patients with sarcopenia had significantly lower BMI values than those without sarcopenia. Additionally, a statistically significant negative correlation was observed between BMI and the SARC-F score in our study.

Handgrip strength has been proposed as a prognostic indicator in chronic liver diseases, complementing assessments of liver functional reserve. Imaging tools such as magnetic resonance imaging (MRI) and computed tomography (CT) are considered gold standards for non-invasive assessment of muscle quantity/mass. However, these instruments are not widely used in primary care due to their high cost, portability challenges, and the need for trained personnel to use them [32]. Algorithms recommending handgrip strength as a sarcopenia screening tool suggest that it may serve as an initial evaluation before confirming sarcopenia using imaging techniques [33]. Handgrip strength offers several advantages: it directly measures muscle function, is cost-effective, is easy to use at the bedside, requires minimal training, and does not expose patients to radiation [34]. As expected, we found that handgrip strength was significantly lower in patients with sarcopenia than in those without.

Serum albumin, primarily synthesized in the liver, plays critical roles in maintaining colloidal osmotic pressure and supporting enzymatic functions. Cirrhotic patients have reduced albumin synthesis and degradation rates compared to healthy individuals [35]. Serum albumin levels, along with bilirubin and INR, are widely used to monitor the nutritional status of cirrhotic patients [36]. Several studies have shown that decreased serum albumin levels are associated with reduced muscle mass and strength, increased sarcopenia risk, and poorer prognosis [18,37]. Consistently with the literature, our study found that patients with sarcopenia had significantly lower serum albumin levels than those without sarcopenia.

Serum ferritin, a sensitive biomarker of iron status in healthy populations, reflects body iron stores and modulates muscle function by influencing mitochondrial function, ferroptosis, and insulin signaling [12]. Ferritin levels are elevated in conditions such as iron overload, inflammation, infections, and liver diseases [38,39]. High ferritin levels have been associated with increased sarcopenia prevalence in previous studies [40,41]. It has been thought that elevated body iron may contribute to oxidative stress and chronic inflammation and that under oxidative stress, elevated serum ferritin may damage skeletal muscle damage by iron overload. Similarly to the studies in the literature, we found that patients with sarcopenia had significantly higher ferritin levels than those without, suggesting abnormal iron distribution rather than decreased iron stores in sarcopenic patients.

The liver plays a crucial role in endogenous vitamin D metabolism. Chronic liver disease patients have lower serum vitamin D levels compared to healthy individuals, with strong associations observed between low vitamin D levels, sarcopenia, and frailty [42,43,44]. Although the mechanism remains unclear, vitamin D receptors are known to exist in skeletal muscle. Experimental models have shown progressive loss of muscle mass and strength in vitamin D deficiency [45]. Consistently with these findings, our study demonstrated that patients with sarcopenia had significantly lower vitamin D levels.

Serum sodium is an independent risk factor for mortality in cirrhotic patients. The inclusion of sodium levels in the MELD-Na score has improved its accuracy in predicting short-term mortality [7,46]. Nakamura et al. reported a strong association between serum sodium levels, sarcopenia, and mortality in cirrhotic patients [47]. It has been thought that hyponatremia in cirrhotic patients is associated with falls, fractures, and poor prognosis. Similarly to the studies in the literature, we also found a statistically significant negative correlation between serum sodium levels and the SARC-F score in our study.

One limitation of our study is the relatively small sample size, necessitating larger studies for more comprehensive analysis.

## 5. Conclusions

This study provides valuable insights into the simultaneous assessment of sarcopenia and frailty in cirrhotic patients, highlighting their associations with handgrip strength and various biochemical parameters. In conclusion, our study demonstrates that sarcopenia and frailty are highly prevalent in this patient population. These findings emphasize the importance of evaluating sarcopenia and frailty in this population, which will be crucial for the management of the disease.

## Figures and Tables

**Table 1:** General characteristics and laboratory values of 100 patients.

Variable	Median (IQR)	Biochemical Data	Median (IQR)
Age, years	66.5 (59–71)	AST (U/L)	52 (28–70)
Gender/Male, n (%)	59 (58.7)	ALT (U/L)	30 (19–47)
BMI (kg/m^2^) (Mean (SD))	26.04 ± 5.43	GGT (U/L)	46(20–75)
Weight loss in the last one year (-kg)	4 (0–5)	ALP	93 (69–150)
CTP, Class-A n (%)	47 (46.7)	Bilirubin (g/dL)	1.5 (1.0–2.2)
Class-B n (%)	28 (28.0)	Creatinine (mg/dL)	0.95 (0.7–1.1)
Class-C n (%)	25(25.3)	Total protein (g/L)	72 (69–77)
CTP score	8 (5–10)	Vitamin D	25.5 (14.0–37.2)
MELD	12 (9–15)	Na (mmol/L)	133 (125–137)
MELD-Na	13 (9–17)	Hemoglobin (g/L)/ Ferritin	12.7 (12.5–13.7)/33 (24–60)
Ascites/Varices n (%)	41.3%/64%	INR for phrothrombrin time	1.3 (1.1–1.5)
Encephalopathy n (%)	32%	Albumin (g/L)	32 (28–42)

**Table 2:** A comparison of the patients based on the sarcopenia status.

Parameters	Non-Sarcopenia (SARC-F < 4) n = 68; 68%	Sarcopenia (SARC-F ≥ 4) n = 32; 32%	*p*-Value
Age, years	66.5 (57–70)	67.5 (60.5–73.8)	0.323
Gender/Male, n (%)	42 (62.7)	16 (50)	0.427
BMI (kg/m^2^)	27.3 ± 4.6	23.2 ± 4.3	<0.001
Weight loss in the last one year (-kg)	2 (0–4)	5.5 (1.5–7.8)	0.004
Etiology of cirrhosis n (%)			
Cryptogenic cirrhosis	14 (21)	7 (20.8)	0.166
NASH	14 (21)	7 (20.8)	
Hepatitis B	13 (18.6)	3 (8.3)	
Alcohol	13 (18.6)	1 (4.2)	
Other factors	14 (21)	14 (45.8)	
Activity level			
Bed bounded	1 (2)	5 (20.8)	<0.001
Physical inability to leave the house	2 (3.9)	11 (45.8)	
Physically able to leave the house	48 (94.1)	8 (33.3)	
CTP score	6 (5–10)	8 (6.0–9.8)	0.029
CTP class			
A	37 (54.9)	9 (29.2)	0.042
B	13 (19.6)	15 (45.8)	
C	18 (25.5)	8 (25.0)	
MELD	10 (8–14)	13.5 (11.0–15.8)	0.069
MELD-Na	10 (9–15)	18 (11.0–21.8)	0.002
Ascites			
No	37 (54.9)	21 (66.7)	0.475
Yes	31 (45.1)	11 (33.3)	
Encephalopathy			
No	49 (72.5)	19 (58.3)	0.413
Under control	15 (21.6)	9 (29.2)	
Uncontrolled	4 (5.9)	4 (12.5)	
Varices			
No	22 (32)	13 (41.6)	0.193
Yes, without bleeding	33 (48)	17 (54.2)	
Yes, with bleeding	13 (20)	2 (4.2)	

BMI: body mass index; CTP: Child–Turcotte–Pugh; NASH: nonalcoholic steatohepatitis; MELD: model for end-stage liver disease.

**Table 3:** A comparison two groups patients of SARC-F, handgrip strength, FRAIL scale status.

Parameters	Non-Sarcopenia (SARC-F < 4) n = 68; 68%	Sarcopenia (SARC-F ≥ 4) n = 32; 32%	*p*-Value
Handgrip strength (kg)			
Right hand	34 (30–44)	25.5 (23.5–29.8)	<0.001
Left hand	35 (17–73)	25 (24–30)	0.001
SARC-F	1 (0–2)	6 (4–7)	<0.001
Strength (How much is the difficulty in lifting 5 kg?)			
No difficulty	51 (74.5)	2 (8.0)	<0.001
Some difficulty	16 (23.5)	15 (46.0)	
A lot of difficulty	1 (2)	15 (46.0)	
Assistance (How much difficulty do you have walking across a room?)			
No difficulty	55 (80.4)	7 (21.0)	<0.001
Some difficulty	13 (19.6)	18 (58.0)	
A lot of difficulty, use aids, or unable to do without personal help	0 (0)	7 (21.0)	
Rise (How much is the difficulty in transferring from a chair or bed, and is the use of aid or help needed?)			
No difficulty	61 (90.2)	9 (29.2)	<0.001
Some difficulty	7 (9.8)	20 (62.5)	
A lot of difficulty, use aids, or unable to do without personal help	0 (0)	3 (8.3)	
Climb (How much is the difficulty of climbing a flight of 10 steps?)			
No difficulty	55 (80.4)	1 (4.2)	<0.001
Some difficulty	12 (17.6)	23 (70.8)	
A lot of difficulty	1 (2.0)	8 (25.0)	
Falls (How many falls have been experienced in the past year?)			
No falls	53 (78.4)	3(8.3)	<0.001
1–3 times falls	15 (21.6)	13 (41.7)	
>3 times	0 (0)	16 (50.0)	
FRAIL Scale			
Non-frail	8 (11.8)	1 (4.0)	0.272
Pre-frailty	43 (62.7)	17 (54.0)	
Frailty	17 (25.5)	14 (42.0)	

**Table 4:** A Comparison of biochemical data of the patients based on the sarcopenia status.

Biochemical Data	Non-Sarcopenia (SARC-F < 4) n = 68; 68%	Sarcopenia (SARC-F ≥ 4) n = 32; 32%	*p*-Value
Albumin (g/L)	38 (29–44)	30 (27–38)	0.004
Bilirubin (g/dL)	1.1 (1.0–1.8)	1.9 (1.4–2.4)	0.01
Creatinine (mg/dL)	0.8 (0.7–1.1)	0.9 (0.5–1.2)	0.168
Total protein (g/L)	74 (69–77)	70.5 (67–74.8)	0.277
AST (U/L)	46 (27–58)	67 (48.5–80.8)	0.029
ALT (U/L)	27 (17–41)	47.5 (24.5–70.0)	0.025
GGT (U/L)	43 (22–80)	37 (15–60)	0.540
ALP (U/L)	91 (73–127)	89 (64.8–203.8)	0.995
INR	1.3 (1.1–1.4)	1.5 (1.1–1.7)	0.383
Vitamin D	32 (19–44)	16 (8–27)	0.008
Na (mmol/L)	136 (131–137)	132 (125–137)	<0.001
Hemoglobin (g/L)	12.1 (105–135)	10 (90.8–129.5)	0.075
Ferritin	35 (23–56)	49 (33.3–66.5)	0.032

ALT: alanine transaminase; ALP: alkaline phosphatase; AST: aspartate aminotransferase; GGT: gamma-glutamyl transferase; INR: international normalized ratio.

**Table 5:** Spearman correlation analysis of various parameters of the patients with SARC-F points.

Parameters	Correlation Coefficient (r_s_)	*p*-Value
Age (years)	0.25	0.032
BMI (kg/m^2^)	−0.43	<0.002
CTP Score	0.271	0.019
Meld Score	0.190	0.098
Meld-Na Score	0.384	0.001
FRAIL Score	0.206	0.072
Handgrip strength (right)	−0.492	<0.001
Handgrip strength (left)	−0.403	<0.001
Albumin (g/L)	−0.344	0.003
Bilirubin (g/dL)	0.336	0.002
Creatinine (mg/dL)	−0.178	0.140
Total protein (g/L)	−0.114	0.331
AST (U/L)	0.249	0.024
ALT (U/L)	0.263	0.024
GGT (U/L)	0.018	0.885
ALP (U/L)	0.021	0.867
INR for phrothrombrin time	0.071	0.551
Vitamin D	−0.400	0.002
Na (mmol/L)	−0.437	<0.001
Hemoglobin (g/L)	−0.212	0.079
Ferritin	0.287	0.013

ALT: alanine transaminase; ALP: alkaline phosphatase; AST: aspartate aminotransferase; BMI: body mass index; CTP: Child–Turcotte–Pugh; GGT: gamma-glutamyl transferase; INR: international normalized ratio; Meld: model for end-stage liver disease. r_s_: Spearman’s rank correlation coefficient; correlation is significant at *p* < 0.05.

## Data Availability

The data presented in this study are available on request from the corresponding author.
